# Targeting protein glycosylation to regulate inflammation in the respiratory tract: novel diagnostic and therapeutic candidates for chronic respiratory diseases

**DOI:** 10.3389/fimmu.2023.1168023

**Published:** 2023-05-15

**Authors:** Xiaofeng Xie, Siyuan Kong, Weiqian Cao

**Affiliations:** ^1^ Shanghai Fifth People’s Hospital and Institutes of Biomedical Sciences, Fudan University, Shanghai, China; ^2^ NHC Key Laboratory of Glycoconjugates Research, Fudan University, Shanghai, China

**Keywords:** protein glycosylation, chronic respiratory diseases, abnormal glycoenzyme activity, glycoproteomics, glycobiomarkers

## Abstract

Protein glycosylation is a widespread posttranslational modification that can impact the function of proteins. Dysregulated protein glycosylation has been linked to several diseases, including chronic respiratory diseases (CRDs). CRDs pose a significant public health threat globally, affecting the airways and other lung structures. Emerging researches suggest that glycosylation plays a significant role in regulating inflammation associated with CRDs. This review offers an overview of the abnormal glycoenzyme activity and corresponding glycosylation changes involved in various CRDs, including chronic obstructive pulmonary disease, asthma, cystic fibrosis, idiopathic pulmonary fibrosis, pulmonary arterial hypertension, non-cystic fibrosis bronchiectasis, and lung cancer. Additionally, this review summarizes recent advances in glycomics and glycoproteomics-based protein glycosylation analysis of CRDs. The potential of glycoenzymes and glycoproteins for clinical use in the diagnosis and treatment of CRDs is also discussed.

## 1 Introduction

Protein glycosylation is a crucial posttranslational modification that affects more than 50% of known proteins. Glycosyltransferases (GTs) and glycoside hydrolases (GHs) are the two main types of glycoenzymes. The Carbohydrate-Active enZYmes Database (CAZy) (http://www.cazy.org) records 116 families of GTs (GT1-GT116) and 173 families of GHs (GH1-GH173), including 235 putative human GTs and 87 putative human GHs. GTs transfer sugar molecules from nucleotide sugar or lipid-linked sugar donors to hydroxyl groups of acceptors to form specific glycan structures and glycosidic linkages (1–3). For instance, sialyltransferases (STs), fucosyltransferases (FUTs), galactosyltransferases (GalTs) and MGAT3 (4, 5) are responsible for the formation of glycans that involve sialic acid (sialylation), fucose (fucosylation), galactose (galactosylation), and bisecting GlcNAc (6, 7), respectively. GHs constitute a superfamily of enzymes that hydrolyze glycosidic linkages during oligosaccharide maturation (3, 8), exhibiting broad and stringent substrate specificities (9). Common GHs include β-glucosidase, glucosidase II, N-acetyl-β-d-glucosaminidase, α-glucosidase, β-galactosidase, β-glucuronidase, α-mannosidase, β-mannosidase, α-fucosidase, and sialidase. O-linked β-N-acetylglucosamine glycosylation (O-GlcNAcylation), a distinctive form of protein glycosylation, involves the dynamic addition of N-acetylglucosamine from UDP-GlcNAc onto specific serine or threonine residues. O-GlcNAcylation cycling is mediated by O-GlcNAc transferase (OGT) and O-GlcNAcase (OGA) (10, 11).

Chronic respiratory diseases (CRDs), affecting the airways and other structures of the lungs, are a major threat to global public health. Common CRDs include chronic obstructive pulmonary disease (COPD), asthma, cystic fibrosis (CF), idiopathic pulmonary fibrosis (IPF), pulmonary arterial hypertension (PAH), non-CF bronchiectasis and lung cancer (12, 13). COPD obstructs the airways, making breathing difficult, while asthma is a chronic inflammatory condition caused by environmental and genetic factors, leading to airways narrowing and excess mucus production during attacks (14). CF is a multisystemic autosomal recessive disease caused by a mutation in the CF transmembrane conductance regulator (CFTR) gene, severely damaging the respiratory and digestive systems (15). Interstitial lung diseases (ILDs), such as IPF (16), are a heterogeneous family of lung disorders characterized by alveolar injury, inflammation, and fibrosis, while PAH is a rapidly progressive disease characterized by elevated pulmonary artery pressure. Non-CF bronchiectasis is a progressive lung disease resulting in permanently dilated airways. Lung cancers can develop from chronic irritation and inflammation (17). Persistent inflammation is a common feature of most CRDs, involving epithelial and immune cells and cytokines in the respiratory tract (18). Proinflammatory cytokines can regulate the glycosylation of cell surface-associated glycoproteins present in immune and epithelial cells (4). Therefore, inflammation-induced glycosylation changes may play a crucial role in the development of CRDs.

Glycoproteins play essential roles in a variety of physiological functions, such as protein folding, cell-cell interaction, cell adhesion, and ligand binding (19). Abnormal protein glycosylation has been observed in the pathological process of CRDs. In this review, we will focus on the role of glycosylation in CRDs by examining the involvement of glycoenzymes and aberrant glycosylation in the pathogenesis of different CRDs from three perspectives: (1) the altered expression of GTs and GHs, along with associated aberrant fucosylation, sialylation, and O-GlcNAcylation in CRDs; (2) advancements in glycomics and glycoproteomics that facilitate the exploration of CRDs; and (3) the potential clinical applications of glycoproteins and glycoenzymes as biomarkers and targets in CRDs.

## 2 Aberrant glycosylation in CRDs

Several experiments have shown that abnormal protein glycosylation is present in almost all CRDs, including COPD (20), asthma (21, 22), CF (23, 24), PAH (25), IPF (26), and lung cancer (27–29). For example, in COPD, an N-glycomics study found an abnormal N-glycosylation pattern in plasma proteins, with a decrease in low branching forms and increase in more complex forms (20). Aberrant fucosylation and sialylation of mucins were observed in asthmatic patients (30, 31). CF airway epithelial cells were characterized by increased fucosylation and decreased sialylation (23). In PAH, augmented expression and activity of OGT, the enzyme for O-GlcNAcylation, were observed, suggesting the role of abnormal O-GlcNAcylation in PAH development (32). Abnormal protein glycosylation is also observed in almost all cancers (33, 34), including lung cancer (27, 28). In IPF, the core fucosylation of TGF-β1, mediated by α-1,6-fucosyltransferase (FUT8), plays a crucial role in the transformation of pericytes into myofibroblasts (35). Changes in the protein glycosylation profile in body fluids, especially serum and sputum, may serve as biomarkers for early diagnosis of CRDs. For example, increased fucosylation levels of serum surfactant protein D (SP-D) were identified as a potential biomarker of COPD (36). Multiple integrated N-glycoproteomics analyses have been performed to screen new effective biomarkers for the early diagnosis of lung cancer and for monitoring lung cancer progression (37–40). Fucosylated alpha-1-acid glycoprotein (AGP), ceruloplasmin (CP), and paraoxonase 1 (PON1) in the serum may be potential biomarkers for lung cancer (41).

Protein glycosylation changes are prevalent in CRDs, making them a promising candidate for early diagnosis and therapeutic targets. Changes in protein glycosylation are often caused by aberrant expression and mutation of glycoenzymes, which affect glycosylation activity. Therefore, the analysis of glycoproteins and their related glycoenzymes has become a widely used approach for screening potential biomarkers and disease-associated regulators in various diseases, including CRDs.

## 3 Glycoenzyme-based protein glycosylation changes in CRDs

CRDs are associated with persistent inflammation in the respiratory tract, where glycoproteins are believed to play a critical role in regulating inflammation-related functions such as cell adhesion, immunogenicity, and cell-to-cell and cell-to-substrate interactions. Proinflammatory cytokines, such as interleukin-1 (IL-1), IL-6, and tumor necrosis factor alpha (TNF-α), are important regulators of inflammation and can influence the expression of GTs, particularly STs and FUTs (42–45). This, in turn, affects downstream protein glycosylation changes, which contribute to the development of CRDs and other chronic inflammation diseases (46).

Various GTs are involved in the biosynthesis of different glycosylation types, and changes in GT expression can alter protein glycosylation patterns. Growing evidence suggests that alteration of GT expression is present in many pathophysiological conditions (19, 47). Dysregulation of GT expression has been observed in multiple cancers (19, 48), such as pancreatic cancer (49), colorectal cancer (50), and breast cancer (51), and it is associated with tumorigenesis, metastasis, and chemoresistance (50). Moreover, a higher frequency of GT variants has been detected in cancers with higher global mutation burdens, as revealed by an integrative pan-cancer analysis (52). Additionally, several GHs are overexpressed in various types of cancer (53), and they play a crucial role in prodrug therapy by activating prodrugs that target cancer and diabetes (53–55). Therefore, GTs and GHs are important factors in physiological and pathological conditions, and their dysregulated expression can lead to various diseases. Abnormal GTs and GHs could serve as potential biomarkers and therapeutic targets.

**Figure 1:**
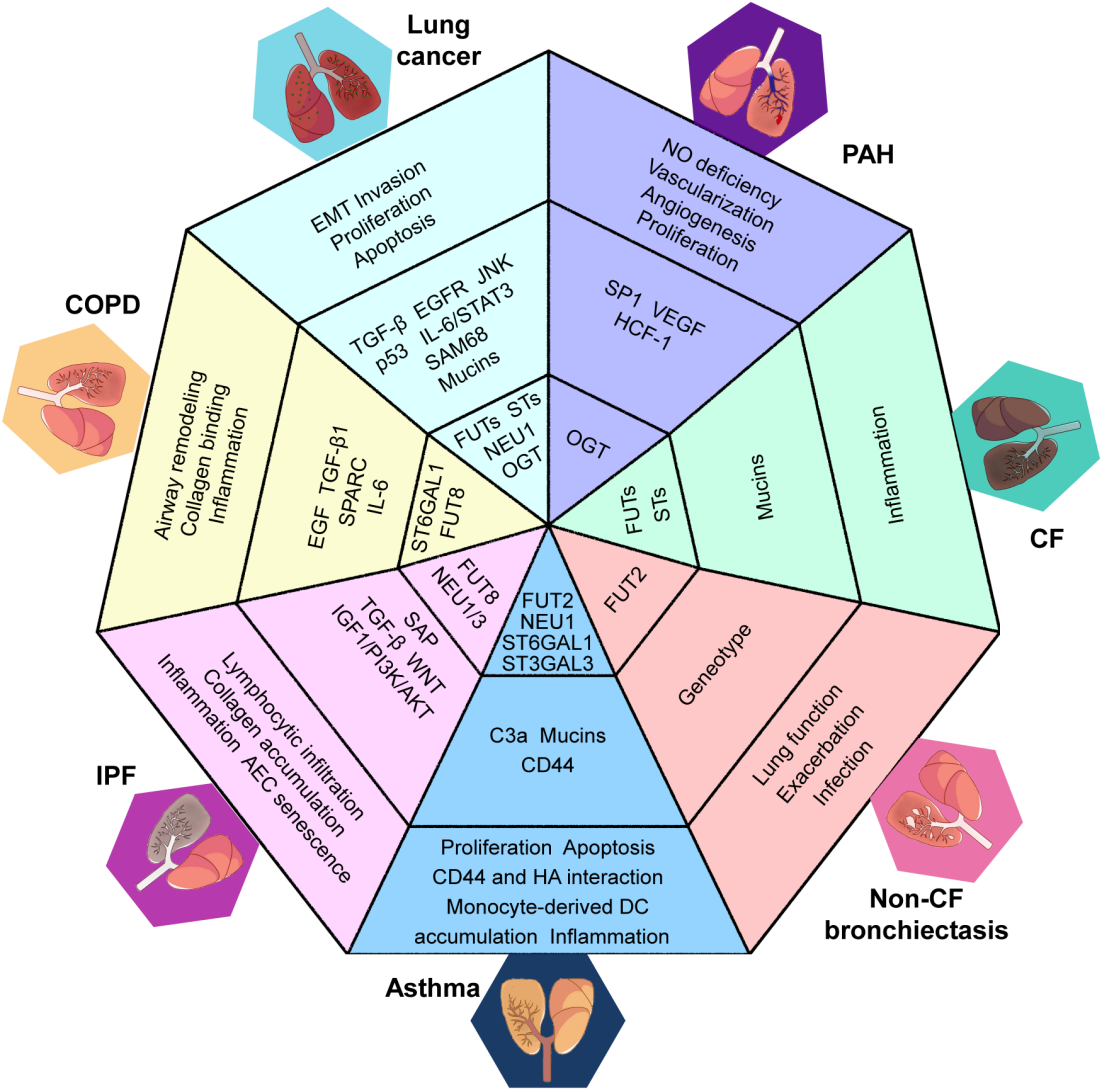
Overview of the glycoenzyme-related protein glycosylation and the functional changes in CRDs. COPD, chronic obstructive pulmonary disease; CF, cystic fibrosis; IPF, idiopathic pulmonary fibrosis; PAH, pulmonary arterial hypertension; EMT, epithelial–mesenchymal transition; ST, sialyltransferase; FUT, fucosyltransferase; O-GlcNAcylation, O-linked β-N-acetylglucosamine glycosylation; OGT, O-GlcNAc transferase; ST6GAL, beta-galactoside alpha-2,6-sialyltransferase; ST3GAL, beta-galactoside alpha-2,3-sialyltransferase; NEU, neuraminidase; TGF, transforming growth factor; EGFR, epidermal growth factor receptor; JNK, c-Jun N-terminal protein kinase; IL, interleukin; STST3, signal transducer and activator of transcription 3; SAM68, SRC-associated in mitosis, 68 kDa; HCF-1, host cell factor-1; NO, nitric oxide; SP1, specificity protein 1; DC, dendritic cells; HA, hyaluronic acid; SAP, serum amyloid P; IGF1/PI3K/AKT, insulin-like growth factor 1/phosphotylinosital 3 kinase/protein kinase B; AEC, alveolar epithelial cell; SPARC, secreted protein acidic and rich in cysteine; EGF, epidermal growth factor.

In CRDs, abnormal fucosylation, sialylation, and O-GlcNAcylation can be attributed to changes in the expression of glycoenzymes, which are responsible for the enzymatic reactions involved in protein glycosylation. The dysregulation of these glycoenzymes is a major factor for underling the aberrant glycosylation patterns observed in these diseases. **Table 1** and **Figure 1** provide a summary of the glycoenzyme-mediated glycosylation changes and associated dysfunction in CRDs. Specifically, we will review the impact of the variations in the expression of FUTs, STs, OGTs and GHs on protein glycosylation in these diseases.

**Table 1:** Altered glycoenzymes and the corresponding changes in CRDs.

CRDs	Glyco-enzymes	Modified key glycoproteins	Role of protein glycosylation in CRDs
COPD	FUT8	SPARC	Decreased core fucosylation of SPARC inhibits collagen binding (56).
	ST6GAL1	IL-6	Reduced ST6GAL1 and α-2-6 sialylation augment IL-6 expression/secretion in human bronchial epithelial cells (57). Plasma ST6GAL1 levels are associated with inflammation and exacerbation of COPD (57, 58).
Asthma	FUT2	MUC5AC	Increased fucosylation of MUC5AC exacerbates airway inflammation and increases mucus viscoelasticity in asthma (59).
	ST6GAL1	MUC4β	Sialylation of MUC4β inhibits epithelial cell proliferation (30).
	ST3GAL3	MUC5B	Sialylation of MUC5B induces apoptosis in eosinophils (59)
	NEU1	CD44	CD44 glycosylation affects its recognition of hyaluronan (60).
CF	FUTs	Mucins	Mutated CFTR may influence the compartmentalization of FUTs (61, 62).
	STs	Mucins and membrane proteins	The sialylation of MUC5B and MUC5AC increases in CF patients’ sputum (61, 63). CFTR ΔF508 mutation decreases membrane sialylation (64).
IPF	FUT3 and FUT5	/	An increase in circulating FUT3 and FUT8 is associated with a reduced risk of IPF (65).
	FUT8	IGF1/PI3K/AKT pathway, TGF-βR, and WNT receptor	Upregulated expression of FUT8 activates IGF1/PI3K/AKT signaling in AEC senescence and IPF (66). Core fucosylation of TGF-βR and WNT receptor activates EMT (67).
	OGT	HCF-1	OGT-facilitated PASMC proliferation through activation of HCF-1 (32)
	NEU1	Collagen types I and III, TGF-β1	Up-regulated NEU1 induces lymphocytic infiltration and increases TGF-β1 and collagen accumulation (68).
	NEU3	SAP and IL-6 and IL-1β	The inhibition activity of SAP on fibrocyte differentiation and IL-10 accumulation reduces in IPF patients with high levels of NEU3 (69). NEU3 upregulates extracellular accumulation of IL-6 and IL-1β (70).
PAH	OGT	HCF-1, SP1, VEGF, and eNOS	OGT facilitates PASMC proliferation through HCF-1 (32). OGT modulates VEGF expression and vascularization in IPAH by regulation of SP1 (32). Increased O-GlcNAcylation of eNOS at site 615 reduces eNOS activity in IPAH (71).
non-CF bronchiectasis	FUT2	/	FUT2 genotype influences exacerbation and infection in non-CF bronchiectasis (72).
NSCLC	FUT1	/	Decreased FUT1 is correlated with low EGFR-TKI responsiveness, poor prognosis and tumor metastasis of NSCLC (73).
	FUT2	TGF-β/Smad signaling, p53 and JNK signaling,	FUT2 facilitates autophagy and suppresses apoptosis *via* p53 and JNK signaling (74). FUT2 promotes EMT by TGF-β/Smad signaling (75).
	FUT4	TGF-β1 and EGFR	FUT4 promotes TGF-β1 secretion and induces EMT (76). Down-regulated FUT4 inhibits EGFR activation, MAPK and NF-κB signal pathways (77).
	FUT8	EGFR	FUT8 regulates the cancer-promoting capacity of cancer-associated fibroblasts by modifying EGFR core fucosylation (78).
	ST3GAL6	EGFR	Downregulated ST3GAL6 regulates EGFR signaling (79).
	ST6GAL1	Jagged1, DLL-1, Notch1, Hes1, Hey1, MMPs and VEGF	Downregulation of ST6GAL1 decreases Jagged1, DLL-1, Notch1, Hes1, Hey1, MMPs and VEGF, and suppresses cancer cell proliferation, migration and invasion (80).
	ST6GALNAc1	MUC5AC	Mutant p53R175H upregulates ST6GalNAc1 expression and the sialyation of MUC5AC, leading to lung cancer metastasis (81).
	ST3GAL4	Membrane proteins	Elevated ST3GAL4 and sialylation of membrane proteins contribute to the activation of E-cadherin/β-catenin, AKT, and ERK/NF-κB mediated signal transduction pathways (82).
	NEU1	/	NEU1 is correlated with the severity of drug resistance in DLKP, a lung cancer model with a series of drug-resistant variants (83). NEU1 is correlated to p53R273H mutation (84).
	NEU3	EGFR	NEU3 overexpression stimulates the ERK pathway through EGFR (85).
	FUCA2	/	High level of FUCA2 expression may contribute to increased infiltration of tumor-associated macrophages and associate with an immunosuppressive microenvironment in pan-cancer (86).
	OGT	SAM68 and p53/c-Myc	High levels of OGT and O-GlcNAcylated SAM68 predict poor prognosis in LUAD (87). Elevated O-GlcNAcylation of p53/c-Myc induces cisplatin resistance (88).
SCLC	OGT	/	Upregulated OGT expression is associated with poor prognosis (89).
Lung cancer	Glucosidase IIβ	P53	Inhibition of glucosidase IIβ decreases activation of the EGFR/RTK and PI3K/AKT signaling pathways in a p53 dependent manner (90).

CRD, chronic respiratory disease; COPD, chronic obstructive pulmonary disease; CF, cystic fibrosis; IPF, idiopathic pulmonary fibrosis; PAH, pulmonary arterial hypertension; NSCLC, nonsmall cell lung cancer; LUAD, lung adenocarcinomas; SCLC, small cell lung cancer; ST, sialyltransferase; FUT, fucosyltransferase; O-GlcNAcylation, O-linked β-N-acetylglucosamine glycosylation; OGT, O-GlcNAc transferase; OGA, O-GlcNAcase; ST6GAL, beta-galactoside alpha-2,6-sialyltransferase; ST3GAL, beta-galactoside alpha-2,3-sialyltransferase; ST6GALNAc, N-acetylgalactosaminide alpha-2, 6-sialyltransferase; NEU, neuraminidase; FUCA2, alpha-L-fucosidase 2; IL, interleukin; HCF-1, host cell factor-1; ECM, extracellular matrix; EMT, epithelial-mesenchymal transition; IGF1, insulin-like growth factor 1; TGF, transforming growth factor; NF-κB, nuclear factor-κB; JNK, c-Jun N-terminal protein kinase; SPARC, secreted protein acidic and rich in cysteine; CFTR, CF transmembrane conductance regulator; MMP, matrix metalloproteinase; VEGF, vascular endothelial growth factor; eNOS, endothelial nitric oxide synthase; EGFR-TKI, epidermal growth factor receptor tyrosine kinase inhibitor; PASMC, pulmonary artery smooth muscle cell; MAPK, mitogen-activated protein kinase; ERK, extracellular-signal-regulated kinase; RTK, receptor tyrosine kinase; PI3K/AKT, phosphotylinosital 3 kinase/protein kinase B; AEC, alveolar epithelial cell; SAM68, SRC-associated in mitosis, 68 kDa; SAP, serum amyloid P; SP1, specificity protein 1./, the modified key glycoproteins were not specified in the associated studies.

### 3.1 Aberrant FUT-mediated fucosylation in CRDs

Fucosylation is a process mediated by 11 N-linked and 2 O-linked fucosylation enzymes, collectively known as FUTs, which include FUT1-11 and protein O-fucosyltransferase 1 and 2 (POFUT1 and POFUT2). This process can be categorized as core fucosylation (α-1,6-fucosylation) or terminal fucosylation (α-1,2-fucosylation and α-1,3/4-fucosylation), depending on the location of fucose in N-glycan. FUT1 and FUT2 are responsible for α-1,2-linkage, while FUT3-7 and FUT9-11 catalyze α-1,3- and α-1,4-fucosylation, and FUT8 participates in core fucosylation. POFUT1 and POFUT2 transfer fucose from GDP-β-L-fucose to serine or threonine residues (41). It is increasingly evident that abnormal fucosylation occurs in cancer and inflammation (91, 92). Aberrant fucosylation of proteins can contribute to tumor proliferation, invasion, metastasis, and immune evasion (93, 94).

#### 3.1.1 COPD

COPD, which encompasses emphysema and chronic bronchitis, is a progressive disease often linked to smoking and a heightened risk for lung cancer, particularly squamous cell carcinoma (95). Studies on COPD animal models and patients have shown that reduced levels of FUT8 and the corresponding reduction in core fucosylation may contribute to the pathogenesis of the disease. Fut8 knockout (Fut8^-/-^) mice exhibited emphysema-like changes in the lung, and Fut8 knockdown (Fut8^+/-^) mice exposed to cigarette smoke were found to be more susceptible to developing emphysema than wild-type mice (96). Knockout of FUT8 also resulted in alveolar destruction and loss of the core-fucosylated secreted protein acidic and rich in cysteine (SPARC), which impairs collagen binding (56). Decreased FUT8 activity has been correlated with poor lung function and exacerbation of COPD in patients (36, 96). FUT8 Thr267Lys mutation is also a risk factor for emphysema (97).

Studies have shown that treating airway smooth muscle cells in a lipopolysaccharide-induced COPD rat model with extracellular matrix components upregulates expression of cytokine factors like TGF-β1 and IL-6, but downregulates matrix metalloproteinase 9 (MMP-9) (98). In contrast, Fut8^-/-^ mice overexpressed MMPs like MMP-12 and MMP-13 and showed a dysfunctional TGF-β1 due to lack of core fucosylation, leading to downregulation of the extracellular matrix (99). As TGF-β1 plays a significant role in lung remodeling (100), the abnormal expression of FUT8 and decline in core fucosylation may affect the cell-to-matrix communication *via* SPARC in COPD (56). Thus, FUT8 is a potential target for developing COPD therapies as it plays an important role in the COPD development and progression through its effects on the core fucosylation of various proteins, including TGF-β1 and SPARC.

#### 3.1.2 Asthma

Asthma is characterized by the hypersecretion of mucus that is primarily composed of glycoproteins. Studies in mice have shown that knockout of the FUT2 gene can reduce eosinophilic inflammation and airway hyperresponsiveness caused by house dust mites, a common trigger for asthma (22). Dysregulation of FUT2 and epithelial fucosylation have been associated with various chronic inflammatory diseases (92). The main component of mucus, MUC5AC, is typically fucosylated, and FUT2 exacerbates asthma through α-1,2-fucosylation of MUC5AC (59). FUT2 genotypes are also linked to asthma risk (101). FUT2 has three secretor genotypes: nonsecretors (homozygous for the loss-of-function of FUT2), heterozygous secretors, and homozygous secretors. Homozygous secretors of FUT2 may have more severe asthma exacerbations and poor lung function (72). Thus, FUT2 and α-1,2-fucosylation may play a crucial role in asthma. Glycosylated immunoglobulin (Ig) affects various allergic diseases, highlighting the importance of Ig glycosylation patterns in mediating allergies, including asthma. Moreover, the glycosylation of IgE, rather than IgG and IgA, has a dominant role in allergy (102).

#### 3.1.3 CF

CF is caused by defective CFTR, affecting chloride channels in mucus and sweat-producing cells. Phenotype analysis of the N-glycosylation of sputum proteins revealed that α-2,6-sialylation and α-1,6-core fucosylation are common structural features present in patients with CF (63). Abnormal glycosylation of mucins, especially MUC5B and MUC5AC, is reported in CF and other pulmonary conditions (61). However, aberrant O-glycosylation and N-glycosylation of mucins may result from bacterial infection and inflammation rather than CF pathogenesis, as both N-sialylation and N-fucosylation of mucins increase (63). In the small intestine of CF mouse models, increased levels of insoluble and soluble fucosylated mucins are observed (103), which may be due to upregulated FUT2 expression. The glycosylation patterns of CF airway epithelial cells alter with increases in α-1,3/4-fucosylation and decreases in α-1,2-fucosylation and sialylation (62). In CF, a different fucosylation phenotype was reported in mucus and airway epithelial cells, with increased α-1,2-fucosylation in mucus (103) and increased α-1,3/4-fucosylation in airway epithelial cells (23), indicating that different FUTs participate in the fucosylation of mucus and airway epithelial cells in asthma.

#### 3.1.4 IPF

The exact etiology of IPF is still unknown, and the five-year survival rate is approximately 45% (104). Progress in improving overall survival in IPF has been limited since 2010 (104). Integrin α-3 (ITA3) mutation A349S has been discovered in ILD, leading to a gain-of-glycosylation (105). This mutation causes lung disorders by disturbing the biosynthesis of ITA3, a highly expressed integrin in lung epithelium that plays a key role in IPF and the epithelial-mesenchymal transition (EMT). The glycosylation of vacuolar H+-adenosine triphosphatase (V-ATPase) may promote collagen degradation and contribute to the progression of IPF (26). FUT8 upregulation is observed in a bleomycin-induced pulmonary fibrosis rat model, while glycyrrhizic acid can alleviate IPF by inhibiting FUT8-mediated core fucosylation of TGF-βR and WNT (67). Patients with IPF show upregulated expression of FUT8 and core fucosylation, which regulate the insulin-like growth factor 1(IGF1) signaling pathway in IPF (66). The upregulated expression of IGF1 is associated with the pathogenesis of IPF (106), and inhibition of core fucosylation alleviates IGF1-induced IPF (106). Therefore, FUT8 and core fucosylation may play a crucial role in the development and deterioration of IPF by regulating multiple signaling pathways, including those for IGF1, TGF-βR, and WNT. Interestingly, lower IPF expression is associated with increased levels of circulating FUT3 and FUT5 (65). Further research is needed to investigate the role of other FUTs in IPF besides FUT8.

#### 3.1.5 Lung cancer

Lung cancer is the leading cause of cancer-related mortality worldwide (107). It can be categorized into non-small cell lung cancer (NSCLC) or small cell lung cancer (SCLC), with NSCLC accounting for approximately 85% of cases and SCLC accounting for 15% (108). Lung adenocarcinoma (LUAD) and lung squamous cell carcinoma (LUSC) are the most common subtypes of NSCLC. A variety of changes in glycosylation have been observed in lung cancer, such as aberrant glycosylation of mucins and increased sialylation of proteins (29), which are potential biomarkers for tumor development and progression (28).

Abnormal protein fucosylation has also been found in lung cancer (41, 109), with FUT8 and POFUT1 proteins being upregulated in blood and tumor tissue of patients with lung cancer (110). These proteins have shown potential as biomarkers for early detection of lung cancer. In addition, the activity of α-1,3-FUTs increases in NSCLC tumor tissues, with upregulated mRNA expressions of FUT3, FUT6, and FUT7 and downregulated mRNA expression of FUT4 (111). Gene expression analysis has revealed that the mRNA expression of FUT2, FUT3, FUT6, and FUT8 are increased in NSCLC, whereas that of FUT1 is decreased (112). FUT1 expression has been correlated with treatment outcomes in LUAD patients receiving epidermal growth factor receptor tyrosine kinase inhibitor (EGFR-TKI) (73). FUT2 is overexpressed in LUAD and may facilitate autophagy and suppress apoptosis *via* the p53 and JNK pathways (74), as well as induce EMT through TGF-β/Smad signaling in LUAD (75). FUT4 expression shows a negative correlation with the overall survival in operable LUAD (113, 114), and may induce lung colonization and distant metastases of lung cancer cells (114). Autophagic tumor-associated macrophages have been found to promote TGF-β1 secretion and EMT in LUAD through the FUT4/p-ezrin pathway (76). Downregulated expression of FUT4 inhibits EGFR activation and MAPK and NF-κB signal pathways, resulting in reduced migration, invasion and EMT (77). FUT4 may also be related to multidrug resistance in lung cancer (115) and may participate in chemoresistance to cisplatin by suppressing FOXO1-induced apoptosis in lung cancer (116). Additionally, FUT4 expression is positively correlated with PD-1 expression, and may be involved in PD-1-mediated-immunosuppression (113). FUT4 and FUT7 may also participate in lung-to-brain metastasis of NSCLC cells (117). Reduced levels of FUT7 CpG DNA methylation have been found in lung cancer, especially in LUSC (118), and FUT7 promotes lung cancer proliferation by activation of the EGFR/AKT/mTOR signal pathway (119). FUT8, whose expression is upregulated in most cancers, regulates the core fucosylation of PD-1 (120, 121), PD-L2 (122), TGF-β (123), TNFR (124), EGFR (78, 125, 126), B7H3 (127), α3β1 integrin (128), and E-cadherin (129) as well as that of mucins (130). Upregulated expression of FUT8 in cancer-associated fibroblasts promotes the construction of an invasive tumor microenvironment in NSCLC through the core fucosylation of EGFR (78). FUT8-mediated core fucosylation of E-cadherin may also be involved in the EMT in lung cancer cells (131). Abnormal FUT8-mediated core fucosylation plays an important role in tumor proliferation, invasion and metastasis, and FUT8 may be a potential biomarker and therapeutic target in lung cancer and many other cancers.

#### 3.1.6 Other CRDs

Studies have reported an increase in glycosylation, particularly in O-GlcNAcylation, in the pulmonary vasculature of idiopathic PAH patients (46, 132). Additionally, there is evidence of a correlation between decreased O-GlcNAc levels and impaired angiogenesis and vascularization in idiopathic PAH (33). The glycosylation of IgG is also elevated in PAH patients (33), suggesting a potential role for glycosylation, especially O-GlcNAcylation, in the pathobiology of PAH.

Non-CF bronchiectasis, a progressive lung disease characterized by permanently dilated airways, may be driven by a complex cycle of infection and bronchial inflammation (133–135). Therefore, breaking this cycle is a promising therapeutic strategy (136). Research has shown that FUT2 polymorphism is closely associated with the prognosis of non-CF bronchiectasis (72). Patients with non-CF bronchiectasis with homozygous secretors of FUT2 had a poorer prognosis compared with those of nonsecretors and heterozygous secretors and exhibited lower lung function, more exacerbation, and a higher frequency of *Pseudomonas aeruginosa*-dominated infection (72). Therefore, FUT2 and α-1,2-fucosylation may serve as potential therapeutic targets for non-CF bronchiectasis.

### 3.2 Aberrant ST-mediated sialylation in CRDs

Cell surface glycoproteins and glycolipids are commonly capped with sialic acids, which are covalently attached through sialylation by four known families of human STs: ST6GAL (ST6GAL1 and 2), ST6GALNAc (ST6GALNAc1-6), ST3GAL (ST3GAL1-6), and ST8SIA (ST8SIA1-6) (109, 132, 137, 138). ST6GAL1 and ST6GAL2 transfer sialic acid to a β-D-galactopyranosyl (Gal) residue (α-2,6-linked sialylation), while ST6GALNAc1-6 transfer sialic acid to a β-D-N-acetylgalactosaminyl (GalNAc) residue (α-2,6-linked sialylation). ST8SIA1-6, one the other hand, creates a linear α-2,8-polysialic acid on various glycoproteins. Different STs show tissue specificity, suggesting distinct sialylation traits in various tissues. Aberrant sialylation, commonly observed in cancer and nervous system diseases (139–141), is also found in the body fluids and tissue of patients with CRDs, especially with lung cancer (109, 142). To gain insight into the role of sialylation in CRDs, we reviewed the abnormal expression of STs and sialylation in CRDs below.

#### 3.2.1 COPD

Research has shown that the sialylation of IgG and other glycoproteins increases in the blood of patients with COPD (143). The glycosylation changes in IgG could potentially serve as a promising biomarker to distinguish between COPD and lung cancer. Furthermore, the level of circulatory ST6GAL1 negatively correlates with the severity of acute airway inflammation, and the administration of recombinant ST6GAL1 to a murine model mimicking acute exacerbations of COPD can alleviate the inflammation symptoms (58). In patients with COPD, low levels of ST6GAL1 and α-2-6 sialylation are associated with poor prognosis, and this may relate to the regulatory effect of IL-6 expression/secretion by ST6GAL1 (57). Therefore, as a potential therapeutic approach, the administration of recombinant ST6GAL1could prevent the exacerbation of COPD.

#### 3.2.2 Asthma

Asthma is categorized as either type 2 (T2) or non-T2 based on the presence of T helper type 2 cells (Th2) and type 2 cytokines (144). The levels of ST6GAL1 and sialylated MUC4β have been shown to be increased in airway specimens from patients with T2 asthma (30), which may cause epithelial dysfunction. In contrast, the level of ST3GAL3-catalyzed sialylation of MUC5B is downregulated in most patients with asthma, and a reduced MUC5B level may be related to the severity of the disease (59). The sialylation pattern of mucins in asthma appears to play an important role in asthma pathogenesis. The sialylation of memory T helper and regulatory T cells alters in asthmatic children and is associated with asthma progression (145). Mice with a hepatocyte-specific knockout of ST6GAL1 (H-cKO) exhibit significantly increased morbidity due to T cell-dependent HDM-induced asthma. ST3GAL3 knockout (St3gal3*^−/−^*) and knockdown (St3gal3*^+/−^*) mice suffer more severe allergic eosinophilic airway inflammation. Therefore, sialylation could serve as a biomarker for the diagnosis and prognosis of asthma. The galactosylation and sialylation at Asn 297 of IgG decreased in the serum of adult allergic offspring from allergic mothers (21), and this reduction of IgG sialylation is associated with proinflammation (146). In summary, protein sialylation is altered in the airways, mucus, T cells, and sera of patients with asthma, and the related STs and sialylated proteins could potentially serve as biomarkers or therapeutic targets.

#### 3.2.3 CF

CF is a hereditary disorder cause by mutations of CFTR, mainly prevalent in white population from Europe, North America, and Australia (147). Approximately 90% of patients with CF have a phenylalanine deletion at codon 508 (ΔF508), which was associated with decreased α-2,3 sialylation on cell membranes (64). Knockout of CFTR (*CFTR^−/−^*) in piglets result in an increase in sialylated mucins in the airways (148). The aberrant sialylation of proteins, especially mucins, is associated with CF. Reduced sulfation and fucosylation and increased sialylation of MUC5B and MUC5AC are also reported in the sputum of patients with CF (61, 63). Bacterial infection can affect mucin O-glycosylation, and protein N-glycosylation in sputum, and the N-sialylation and N-fucosylation of sputum proteins were increased in these patients (63). The proinflammatory cytokine TNF induces the expression of ST3GAL4 in lung epithelial cells (149, 150), and the inflammatory cytokines IL-6 and IL-8 upregulate the expressions of ST6GAL2 and ST3GAL6 in CF epithelial cells (151), indicating that STs may play a crucial role in inflammation conditions, including CF.

#### 3.2.4 Lung cancer

Aberrant sialylation is a common feature in various cancers, including colorectal cancer (152), ovarian cancer (153), prostate cancer (153), and lung cancer (142). Sialylation is known to be involved in the regulation of tumor metastasis, cell survival, immune evasion, and multidrug resistance (140). A study on lung tumor N-glycoproteomics showed that 108 out of 303 quantified sialylated N-glycopeptides were differentially expressed, and differential Ig α-2,6-sialylation was also observed in lung tumor tissues (142). Moreover, hypersialylation and hyperfucosylation of saliva glycoproteins may be a hallmark of lung cancer in patients (109). The expressions of ST3GAL1, ST6GALNAc3, and ST8SIA6 are significantly reduced in lung cancer tissues and cells, whereas that of ST6GAL1 is significantly upregulated (80). ST6GAL1 may be involved in cell invasion and tumorigenesis of NSCLC *via* Notch1/Hes1/MMP signaling (80). ST3GAL4 may regulate the proliferation, invasion, and migration of NSCLC cells through α-2,3 sialylation of HSP60 (82). ST3GAL6 expression is downregulated in LUAD, which results in poor prognosis (79). Trp53^R172H^ mutation increases the expression of ST6GALNAc1, which may promote tumor metastasis through sialylation of MUC5AC in LUAD (81). Thus, various aberrant ST expression profiles contribute to the abnormal sialylation patterns in lung cancer, which may partially contribute to its development and metastasis.

### 3.3 Aberrant OGT and OGA-mediated O-glycosylation in CRDs

According to the Human Protein Atlas database (https://www.proteinatlas.org/), the expression of OGT is most prominent in the respiratory system compared to other organs, while OGA expression is average. The OGT/OGA pairs may actively work in the regulation of the respiratory system. O-GlcNAcylation participates in various fundamental cellular processes, including transcription, epigenetics, cell signaling dynamics, protein translation, stability, and turnover (10, 154). Dysfunction in O-GlcNAcylation is involved in several diseases, including neurodegenerative diseases (155, 156), diabetes (157, 158), and cancers (159, 160). Therefore, targeting O-GlcNAcylation could be critical in developing new treatments for these diseases (161), especially cancers (162).

#### 3.3.1 PAH

Glycosylation, especially O-GlcNAcylation, increases in the pulmonary vasculature of patients with idiopathic PAH (IPAH) (32, 163), while decreased O-GlcNAc levels correlate with impaired angiogenesis and vascularization in IPAH (25), suggesting that O-GlcNAcylation may contribute to the pathobiology of PAH. OGT is overexpressed in the pulmonary vasculature of patients with IPAH, and its levels are negatively associated with the severity and prognosis of IPAH (32). In IPAH, the OGT/OGA pairs regulates cell proliferation by mediating the activation of host cell factor-1 (HCF-1) in IPAH (32) and can regulate the expression of vascular endothelial growth factor (VEGF) by O-GlcNAcylation of specificity protein 1 (SP1) (164). Nitric oxide (NO) deficiency is also implicated in the development PAH (165), as endothelial NO (eNOS) activity, which produces NO, is reduced by O-GlcNAcylation at residue 615 in PAH (71). Thus, targeting OGT may be a promising for the diagnosis and treatment of PAH.

#### 3.3.2 Lung cancer

In lung cancer, O-GlcNAcylation and expression of OGT are increased, potentially promoting tumorigenesis and cancer progression (166). O-GlcNAcylation can accelerate the Kras^G12D^-induced lung tumorigenesis (167). The nuclear RNA-binding protein, SAM68, has 11 identified O-GlcNAcylation sites (87). High levels of OGT and SAM68 result in poor prognosis of LUAD, and O-GlcNAcylated SAM68 may participate in modulating lung cancer aggressiveness (87). Overexpression of IL-8 can enhance protein O-GlcNAcylation, which may play an important role in the generation and maintenance of cancer stem cells in lung cancer (168). Elevated O-GlcNAcylation of p53/c-Myc induces cisplatin resistance, which could be an important mechanism of drug resistance (88). Upregulation of OGT is observed in SCLC and is associated with clinical outcomes of SCLC (89). OGT and O-GlcNAcylation may play a key role in the IL-6/STAT3 signaling-induced migration and invasion of lung cancer (169). altogether, OGT is typically overexpressed in lung cancer, resulting in hyper-O-GlcNAcylation of tumorigenesis and metastatic-related proteins. OGT has potential as a biomarker and drug target for lung cancer.

### 3.4 Aberrant GHs in CRDs

In addition to GTs, dysregulated expression of GHs can also contribute to the aberrant glycosylation in various diseases, especially in cancer (37, 38). GHs have been used in prodrug activation for cancer and diabetes treatment (38, 39). Interestingly, a higher incidence of congenital disorders of GHs has been observed due to the prevalence mutations in several GH genes in comparison to that in GT genes (40). GHs not only serve as critical enzymes in glycosylation but also play an essential role in prodrug design. The human sialidase family, also referred to as neuraminidases (NEUs), includes NEU1, NEU2, NEU3, and NEU4 (170). Among them, NEU1 is the predominant sialidase expressed in human airway epithelia and lung microvascular endothelia, which can inhibit endothelial cell migration (171, 172). Additionally, NEU1 has been implicated in various airway epithelia- and microvascular endothelia-related inflammatory reactions and diseases. NEU3 is also expressed in human airway epithelia and lung microvascular endothelia (171, 173).

#### 3.4.1 IPF

NEU1 and NEU3 are the predominant sialidases in the lung microvascular endothelia (171). Increased expression of NEU1 is observed in the lungs of patients with IPF, and NEU1 may participate in the IPF pathogenesis by provoking lymphocytic infiltration and promoting accumulation of glycoprotein TGF-β1, type I and III collagen (68, 174). In mice, administration of NEU3 has been shown to induce lung fibrosis, with a gender-specific effect (175). NEU3 can stimulate extracellular accumulation of profibrotic cytokines IL-6 and IL-1β, and conversely, IL-6 can induce the expression of NEU3 in human peripheral blood mononuclear cells (70). Sialylation of serum amyloid P (SAP) is critical for its biological activity. It can inhibit the differentiation of monocytes into fibrocytes and promote high extracellular levels of IL-10. However, in patients with IPF, the sialidase NEU3 is highly expressed, leading to SAP desialylation in the sera (69). This desialylation may contribute to the dysregulated immune response and fibrotic remodeling observed in IPF. Maintaining SAP sialylation status could be a potential therapeutic strategy for modulating the pathogenesis of IPF. Therefore, inhibiting NEU1 and NEU3 could be a new therapeutic strategy for IPF.

#### 3.4.2 Lung cancer

The abnormal expression of certain GHs has been observed in lung cancer. For instance, β-glucuronidase is preferentially concentrated within areas of necrosis in lung tumor tissues (176). Glucosidase IIβ subunits are overexpressed in lung tumor tissues and promote cell growth and migration through receptor tyrosine kinase (RTK) signaling and the p53 pathway (177, 178). Inhibition of these subunits can induce autophagy and apoptosis in lung cancer cells (90). The expression of NEU1 is increased in NSCLC tumors with p53^R273H^ mutation and is associated with poor prognosis (84). NEU1 is also correlated with the severity of drug resistance in DLKP, a lung cancer cell line (83). NEU3 may regulate the ERK pathway *via* EGFR and serve as a prognosis biomarker for EGFR-targeted therapies in NSCLC (85). Furthermore, FUCA2, a fucosidase, is upregulated in most tumors and predicts poor overall survival in pan-cancer, including LUAD (86). These observations suggest that several GHs may serve as potential biomarkers or drug targets for lung cancer.

#### 3.4.3 Other CRDs

There is a known correlation between diabetes mellitus (DM) and COPD, and studies have shown that patients with DM taking α-glucosidase inhibitor drugs have a higher morbidity of COPD (179, 180). In pediatric allergic asthma, the downregulation of NEU1 has been observed in the airway epithelial cells (181). In asthmatic mouse models, the interaction between hyaluronan and CD44 is crucial for the accumulation of antigen-specific Th2 cells (60, 182). NEU1 may also contribute to Th2 cell-mediated airway inflammation by influencing the glycosylation of CD44 (60, 182). Additional, abnormal activity levels of alpha-fucosidase, alpha-galactosidase, beta-galactosidase, alpha-glucosidase, beta-glucosidase, beta-glucuronidase, beta-hexosaminidase, and alpha-mannosidase have been were detected in the sera of patients with CF (183).

### 3.5 Summary

Bacterial infection and cigarette smoking are two major risk factors for CRDs. Smoke disrupts the heavily O-glycosylated MUC1 barrier that protects the airway, and N-acetyl-galactosaminyl transferase-6 (GALNT6), an enzyme that mediates the initial step of O-glycosylation, may be involved in the smoking-induced aberrant MUC1 glycosylation (184). Frequent respiratory infection is a symptom of many CRDs, such as COPD, CF, and non-CF bronchiectasis. Infection may change the glycosylation of lung epithelium cells, such as upregulation sialylation during *Mycobacterium tuberculosis* infection (185). Abnormal MUC1 glycosylation may be the major cause of persistent infection. Moreover, gender biases characterize most chronic inflammation diseases of airway (186, 187), including COPD (188, 189), asthma (190), CF (191), non-CF bronchiectasis (192),and lung cancer (188). Women usually suffer more severe symptoms and have a poorer prognosis for CRDs than men (187). One reason for this may be estrogen regulation of protein glycosylation by affecting the expressions of glycoenzymes. Estradiol can increase FUT4, FUT5, and FUT6 expressions as well as the total amount of fucosylation (193). Estrogen can affect the expressions of ST3GAL1 (194), ST6GAL1 (195), and ST6GAL3 (196), indicating a regulation of sialylation by estrogen. Therefore, estrogen may be a mediator in the progression of CRD.

In conclusion, alterations in protein glycosylation mediated by GTs play a crucial role in the pathogenesis of different CRDs. Various cytokines, such as TNF-α and IL-6, may participate in the regulation of GT expression leading to proinflammatory responses. Additionally, downstream regulatory molecules like TGF-β1 and mucins, which are glycoproteins, contribute significantly to the development of CRDs. The expression profile of GTs in CRDs is also affected by factors such as infection, smoking, and gender. Our review of glycoenzyme-mediated glycosylation changes in CRDs highlights the potential for identifying biomarkers and drug targets of various CRDs.

## 4 Glycomics and glycoproteomics-based protein glycosylation changes discovered in CRDs

Traditional methods in molecular biology for screening target markers (28, 143, 145) and investigating physiological and pathological phenomena (36, 96, 97) often rely on studying the function and mechanism of one or a few proteins in tightly controlled experimental systems. However, such methods have limitations when it comes to analyzing large-scale samples and comprehensively capturing the complexity of biological systems. By contrast, omics strategies can provide a more inclusive molecular perspective of biological systems by analyzing diverse biological and clinical samples, which can lead to the identification of more effective biomarkers and a more comprehensive understanding of the underlying physiological and pathological mechanisms.

In recent years, glycomics and glycoproteomics strategies have been increasingly employed to study the protein glycosylation under various physiological and pathological conditions (197–199). Glycoproteomic analysis characterizes glycopeptides and provides information on glycoforms and their occupation sites (200), while glycomics profiles glycans, enabling the characterization of glycan structures and isomers (200, 201). These complementary strategies offer a comprehensive understanding of total glycosylation patterns. Lectin array and high-performance liquid chromatography (HPLC) with optical detection, commonly used for glycosylation analysis in respiratory-related diseases, have limited capabilities for glycan structure characterization (200–202). Mass spectrometry (MS)-based glycomics and glycoproteomics strategies have proven to be powerful for global glycosylation analysis in complex and large-scale biological samples (203–205) (206, 207). This section reviews recent developments in glycomics and glycoproteomics for the analysis of protein glycosylation in CRDs.

### 4.1 The MS-based glycomics analysis in CRDs

In recent years, there has been a growing interest in protein glycosylation and the use of MS technology for glycomics analysis in CRDs. A typical MS-based glycome experimental procedure involves sample pretreatment, glycan release, glycan purification and separation, glycan derivatization, MS analysis, and data interpretation. Efficient release of glycans from glycoproteins is a critical initial step in glycome analysis, which can be achieved by enzymes (e.g., peptide N-glycosidase F, peptide N-glycosidase A, endo-β-acetylglucosaminidase F, and O-glycosidases) or chemical methods (e.g., hydrazinolysis, β-elimination, and oxidation strategies) (201). MS-based characterization of released glycans can be performed in their native state or after chemical derivatization. Glycan derivatization can be achieved through derivation of the reducing end, hydroxyl group, and carboxyl group (201). For example, phenylhydrazine labeling (a reducing end derivation method) has been used for structural studies of fucosylated N-glycans by MALDI-MS in positive ion mode (208), and for efficient detection and discrimination of SA linkages when following alkyl esterification (209). MS-based systems commonly used for glycomic characterization include MALDI, electrospray ionization (ESI), capillary electrophoresis (CE), gas chromatography (GC) and liquid chromatography (LC) coupled to MS (143).

**Figure 2:**
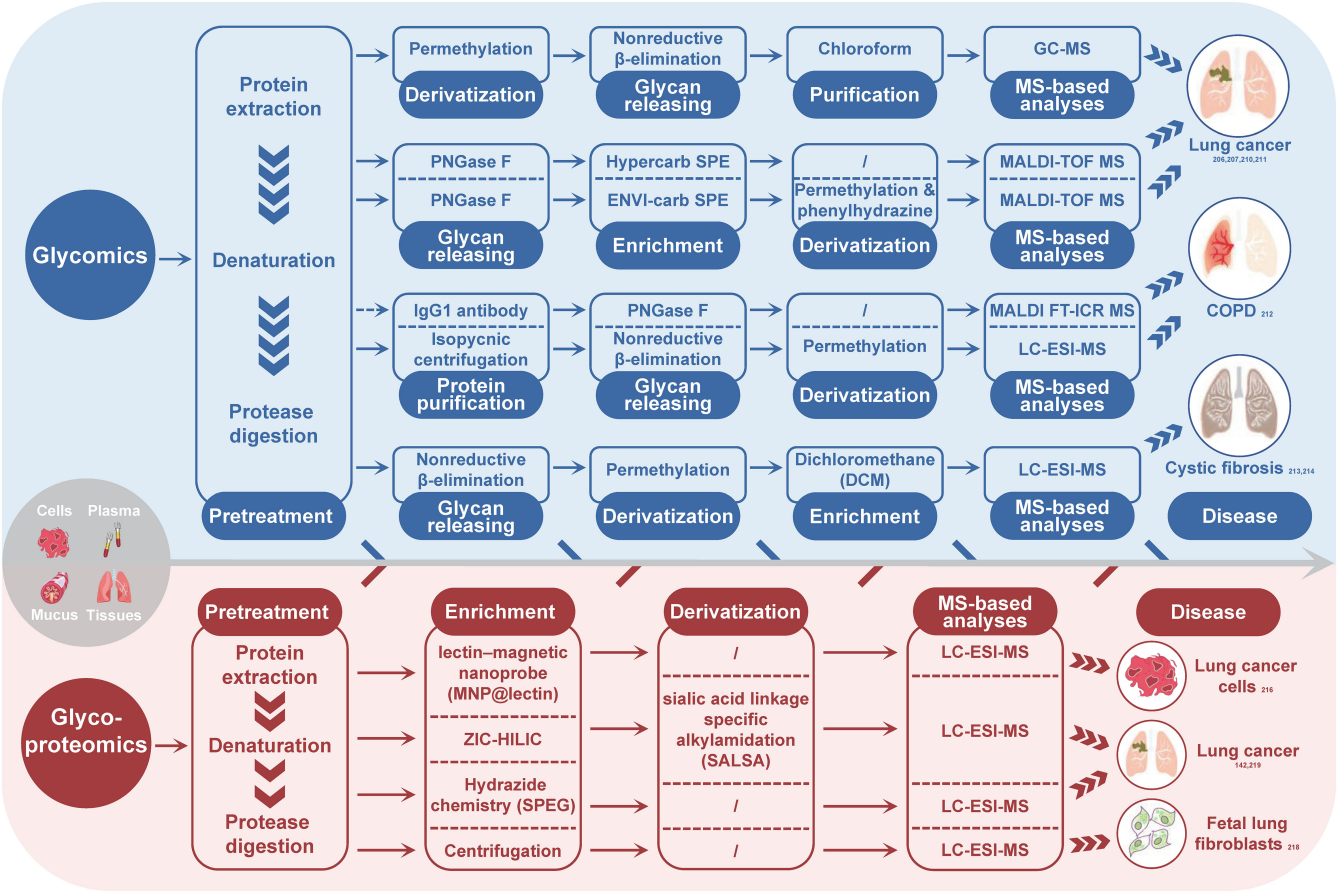
Summary of MS-based glycomics and glycoproteomics strategies used for glycosylation analysis in CRDs. The subscript number indicates the corresponding reference. PNGase F, peptide-N-glycosidase F; SPE, solid phase extraction; MS, mass spectrometry; GC-MS, gas chromatography-mass spectrometry; MALDI-TOF-MS, matrix-assisted laser desorption/ionization-time of flight mass spectrometry; MALDI FT-ICR MS, matrix-assisted laser desorption/ionization fourier transform-ion cyclotron resonance mass spectrometry; LC-ESI-MS, liquid chromatography-electronic spray ionization-mass spectrometry.

In the field of respiratory-related diseases, such as lung cancer, COPD, CF, and asthma, glycomics has gained increasing attention in recent years. Researchers have identified potential biomarkers for the diagnosis and differentiation of these diseases by studying fucosylated, sialylated, and galactosylated glycans with different structures. Borges’ research group (206, 207) found that α-2-6 sialylation, β-1-4 branching, β-1-6 branching, terminal, core and outer arm fucosylation markers were most effective in discriminating between lung cancer cases and controls, with the diagnostic performance being dependent on the cancer stage. Ma et al. (210) reported an increase in the abundance of fucosylated N-glycans from 40.9% to 48.3% in LUSC. Lattová et al. (211) reported a decrease in the amount of double-terminal core fucosylated glycans of the sialylation complex in LUAD. McQuiston et al. (212) found a significantly increased IgG1 N-glycan profile in lung transplant recipients with COPD and primary graft dysfunction. Glycomic analysis of mucins in CF revealed a significantly high abundance of sialylated glycans, and the total abundance of nonsulfated O-glycans correlated with the relative abundance of pathogens (213), whereas another study reported that submucosal gland mucins contained shorter and less branched glycans (214). The glycomics strategies utilized in the CRDs cases are concisely outline in **Figure 2** (blue part).

Glycomics enables the analysis of glycan phenotypes and provides information on the levels of different glycan subtype and associated glycoenzymens. In particular, glycan subtyping analysis can reveal the activity and expression of glycoenzymes, making it a valuable strategy for studying glycoenzyme-mediated functions. Studies on the human lung N-glycome have shown that biantennary complex-type N-glycans dominate (215), but dysregulation of the glycosylation-regulating mechanisms in CRDs can lead to changes in glycan structures, such as increased fucosylation (including terminal, core, and outer arm fucosylation), sialylation, and branching. The core fucosylation depends on FUT8 activity, while outer arm fucosylation relies on FUT3, FUT5, FUT6, and FUT11. ST6GalI glycosyltransferase is responsible for α-2-6 sialylation, and β-1-4 and β-1-6 branching of N-glycans are regulated by GnT-IVa and GnT-V enzymes, respectively (206, 207). However, because glycans can vary greatly in their length, branching, and connectivity, software for interpreting MS data from polysaccharides often requires manual intervention. The lack of generic annotation software has hindered the extensive investigation of the glycome in large scale sample sets from diverse disease, including the field of CRDs.

### 4.2 The MS-based glycoproteomics analysis in CRDs

Glycoproteomics is a valuable strategy for identifying potential biomarkers and understanding physiological and pathological changes. Unlike glycomics, a typical experimental procedure of glycoproteomics involves protein digestion, glycopeptide enrichment, MS analysis and MS data interpretation by software tools (142, 216, 217). Glycopeptide enrichment is critical for efficient glycopeptide identification, and different enrichment methods have been developed, such as lectin enrichment, hydrophilic interaction chromatography, boronic chemistry, hydrazide chemistry, reductive amination chemistry, oxime click chemistry, and ultracentrifugation (218). Although MS-based site-specific glycosylation analysis methods have developed rapidly in recent years, only a few glycoproteomics studies on the intact glycopeptide level have been conducted in the context of CRDs, mainly on lung cancer and pulmonary fibroblasts. For instance, Waniwan et al. (216) developed a lectin-magnetic nanoprobe for glycopeptide enrichment and site-specific glycosylation analysis and identified over 2,000 glycopeptides in NSCLC cell lines. Yang et al. (142) conducted an N-glycoproteomic study using selective alkylamidation and multiple tandem mass tag (TMT)-tagged sialic acid linkages to specifically quantify glycoproteins in lung cancer tissues. Yang et al. (219) found that the expressions of 11 glycoproteins were upregulated in both LUAD and LUSC, while two glycoproteins (ELANE and IGFBP3) and six glycoproteins (ACAN, LAMC2, THBS1, LTBP1, PSAP, and COL1A2) were increased in either LUSC or LUAD. The most comprehensive study on lung fibroblasts was published by Takakura et al. in 2015 (218). They used glycoproteomics method to analyze the membrane fraction of human fetal lung fibroblasts and identified more than 272 glycoforms on 63 sites of 44 glycoproteins. **Figure 2** (red part) summarizes the glycoproteoimcs strategies applied in the CRDs cases.

Although glycoproteomics is a valuable strategy for studying the physiology and pathology of diseases and discovering biomarkers, it has been rarely used in the field of CRDs, and there has been no large-scale clinical sample data output yet. Actually, in the field of glycoproteomics, several relatively mature software portfolios have been established for identification and quantification, such as pGlyco3 (220, 221) and pGlycoQuant (220, 221). These tools are state-of-the-art for site-specific glycome analysis, providing fast and precise identification and quantification of intact glycopeptides. Moreover, high-throughput glycopeptide enrichment technologies for large-scale samples are also available, such as the one developed by Jiang et al. (222), which allows for high-specificity and high-throughput glycopeptide parallel enrichment in a 96-well plate. With the emergence of more effective methods and accurate identification and quantitative tools in the field of glycoproteomics, it is expected that research on glycosylation in CRDs will reach new levels.

## 5 Clinical potential in therapeutic applications

Protein glycosylation remodeling is a common feature of several pathological conditions, resulting from the dysregulated expression of glycoenzymes. Evaluating the expressions of glycoenzymes and protein glycosylation patterns could be a promising approach to creating diagnostic and prognostic biomarkers. Furthermore, targeting the dysregulated glycoenzymes and their associated changes could serve as a potential therapeutic strategy.

### 5.1 Potential diagnostic biomarkers

Protein glycosylation patterns vary across different CRDs due to the diverse profile of glycoenzymes (223, 224). Saliva and serum proteins with abnormal glycosylation have been identified as potential biomarkers for the diagnosis and prognosis of various CRDs (109, 225). Sputum proteins in progressive CRDs, such as COPD and CF, have also been observed to have aberrant glycosylation (63). **Table 2** provides an overview of potential glycoprotein and glycoenzyme biomarkers.

**Table 2:** Potential clinical applications of glycoenzymes and glycoprotein as biomarkers in CRDs.

Disease	Biomarkers	Clinical applications	Reference
COPD	Serum SP-D fucosylation levels	Diagnosis	(36)
COPD	Serum ST6GAL1	Prognosis	(57)
COPD	Sputum glycosylated BPIFB1	Prognosis	(226)
Asthma	Serum IgG glycosylation patterns	Offspring asthma prognosis	(21)
IPF	Serum FUT3	Diagnosis	(21)
IPF	Serum NEU3 and sialylated SAP	Diagnosis and prognosis	(69)
non-CF bronchiectasis	FUT2 genotype	Diagnosis and prognosis	(72)
NSCLC	Tumor FUT1	Prognosis	(73)
	Tumor FUT2	Prognosis	(74, 75)
	Tumor FUT3	Diagnosis and prognosis	(111)
	Tumor FUT4	Prognosis	(113, 117, 227)
	Tumor FUT7	Prognosis	(117)
	Tumor and Serum FUT8	Diagnosis and prognosis	(78, 110)
	Tumor and Serum POFUT1	Diagnosis	(110)
	Tumor ST3GAL1, ST6GALNAc3, ST8SIA6 and ST6GAL1	Prognosis	(80)
	Tumor ST6GALNAc1	Prognosis	(81)
	Tumor ST3GAL6	Prognosis	(79)
	Tumor NEU3	Prognosis of EGFR targeted therapies	(85)
SCLC	Tumor OGT	Prognosis	(89)

COPD, chronic obstructive pulmonary disease; SP-D, surfactant protein-D; ST6GAL, beta-galactoside alpha-2,6-sialyltransferase; BPIFB1, bactericidal/permeability-increasing fold-containing protein B1; IgG, immunoglobulin G; IPF, idiopathic pulmonary fibrosis; FUT, fucosyltransferase; NEU, neuraminidase; SAP, serum amyloid P; CF, cystic fibrosis; NSCLC, nonsmall cell lung cancer; POFUT1, protein O-fucosyltransferase 1; ST3GAL, beta-galactoside alpha-2,3-sialyltransferase; ST6GALNAc, N-acetylgalactosaminide alpha-2, 6-sialyltransferase; ST6GAL, beta-galactoside alpha-2,6-sialyltransferase; SCLC, small cell lung cancer; EGFR, epidermal growth factor receptor; OGT, O-GlcNAc transferase.

For COPD, serum fucosylation levels of SP-D may serve as a diagnostic biomarker (36), while serum levels of ST6GAL1 may predict acute exacerbation of the disease (57). Glycosylated BPIFB1 in sputum may also act as a prognostic biomarker for COPD in smokers (226). In maternal asthma during pregnancy, the IgG glycosylation patterns may predict offspring asthma susceptibility (21). In IPF, circulating FUT3 levels are negatively associated with the risk of the disease and may serve as a biomarker. NEU3 may be involved in the IPF pathogenesis and a drug target, and serum NEU3 and sialylated SAP may act as biomarkers for IPF diagnosis and prognosis (69). FUT2 genotype in patients with non-CF bronchiectasis may also predict the disease outcomes (72). In lung cancer, various FUTs and STs expressed in tumor tissues have been identified as potential diagnostic and prognostic biomarkers (**Tables 1**, **2**) (111, 112). Additionally, NEU3 is a promising biomarker for evaluating EGFR-targeted therapies in patients with NSCLC (85), and the expression level of GTs in lung cancer tumors may act as a biomarker for the diagnosis, prognosis, and treatment assessment (**Table 2**).

**Figure 3:**
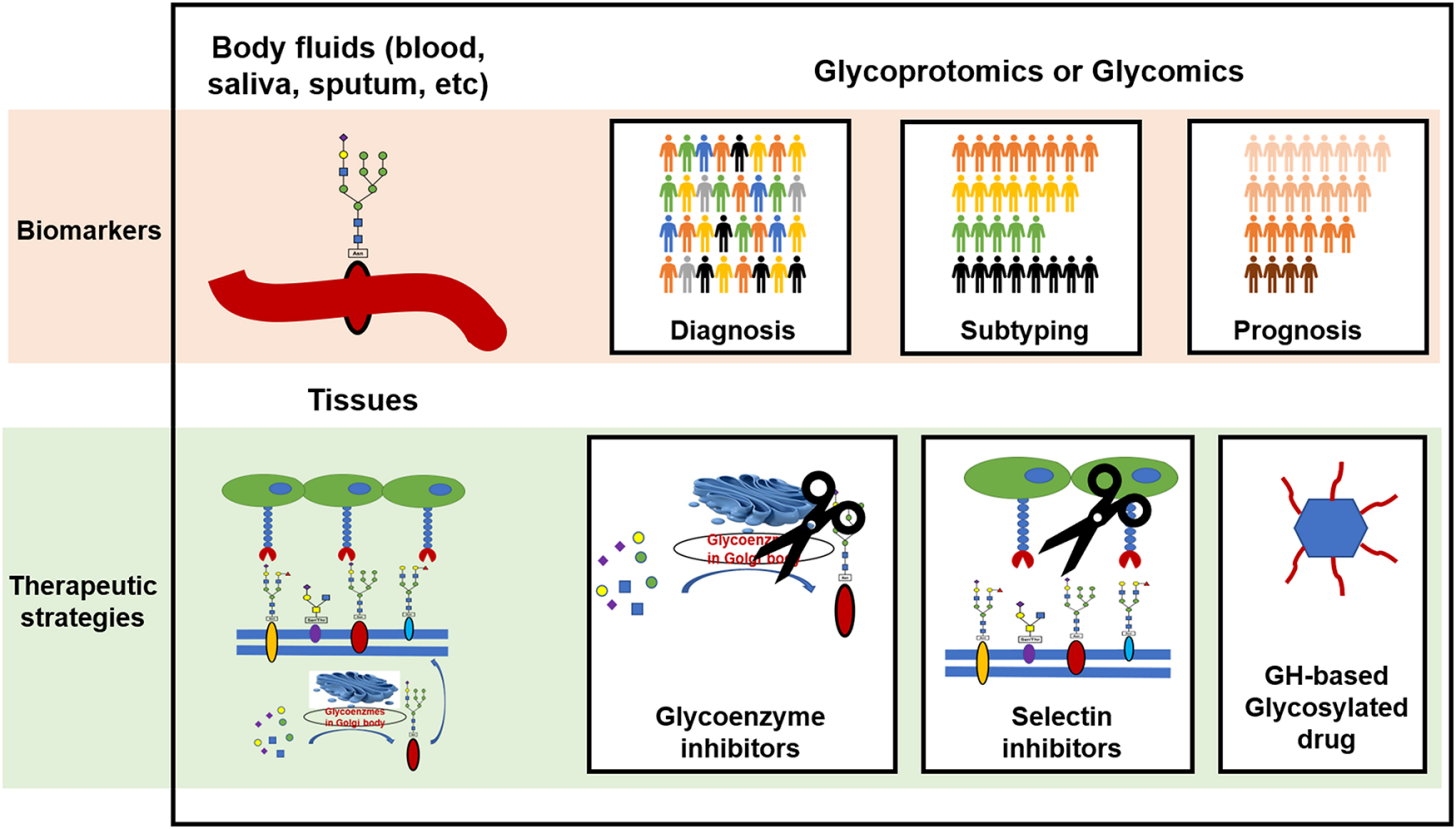
The clinical potential of protein glycosylation for the development of biomarkers and therapeutic strategies in CRDs. GH, glycoside hydrolase.

Glycoproteomics and glycomics are highly valuable strategies for the comprehensive analysis of glycoproteins and glycans in body fluids. By comparing the glycosylation profile of healthy and diseased individuals, researchers can identify specific changes in glycosylation patterns that are associated with particular diseases. This can be used to develop biomarkers for disease diagnosis, drug selection, and prognosis prediction, and to subtype patients and evaluate disease severity based on glycosylation patterns as illustrated in **Figure 3**. These techniques are also useful for monitoring the effects of therapy.

### 5.2 Therapeutic strategies

Aberrant protein glycosylation may serve as both a cause and a consequence of CRDs. These changes in glycosylation can influence the functions of the glycoproteins. Potential therapeutic strategies for CRDs include targeting glycoenzymes to correct the protein glycosylation status, blocking abnormal selectin-mediated cell-cell interactions, and clearing dysfunctional glycans as depicted in **Figure 3**.

One promising approach for treat diseases involves using specific glycoenzyme inhibitors. Aberrant expression of GTs and the related changes in glycosylation are reviewed in the second section of this review. Increased expression of FUTs and STs is characteristic of most patients with NSCLC, and patient-specific overexpressed FUTs and STs are promising targets for developing new therapies. Munkley J. reviewed the inhibitors of STs, including ST3GAL1, ST3GAL3, ST6GAL1, ST6GalNAc2, and ST8SIA3 (140). Increased expressions of ST3GAL4 (82), ST6GAL1 (80), and ST6GalNAc1 (81) were identified in most patients with NSCLC, and inhibiting these enzymes may suppress NSCLC cell metastasis. Further research is required to map the expression of all STs in various lung cancers, and the use of different ST inhibitors in combination therapy may be a new promising cancer therapy.

Overexpression of most FUTs, including FUT2-8, has been observed in lung cancers, especially NSCLC (78, 112, 114, 117, 228). *In vitro* studies have demonstrated that inhibiting or genetically depleting FUT2 (228) and FUT4 (114) can be effective therapies for these cancers. FUT8, a key regulator of the p53 signaling cascade, is a promising therapeutic target for cancer and inflammatory diseases, including Alzheimer’s disease (AD) (229). FUT8 and core fucosylation inhibition are prospective therapeutic strategies for cancer and inflammation. In addition to inhibitors, glycomimetics may also offer alternative therapeutic strategies. Keratan sulfate disaccharide L4 and derivatives show promise as potential drugs for treating emphysema and COPD (230).

Selectins, which are transmembrane glycoproteins found on endothelial cells (E-selectin), leukocytes (L-selectin) and platelets (P-selectin), play a critical role in mediating leucocyte-endothelial adhesion during inflammatory and immune reactions associated with tumorigenesis and metastasis (231). Blocking selectin-ligand interaction interactions is being investigated as an anti-metastasis therapy. P-selectin (232) and E-selectin (111) may be involved in NSCLC cell metastasis, and disrupting the selectin-ligand interactions could potentially serve as a complementary therapy to traditional anticancer therapy (140). Furthermore, selectin antagonists are explored as potential drug candidates for other respiratory inflammatory conditions, such as CF, asthma and COPD (23, 233, 234). Targeting selectins has the potential to be a promising immunomodulation intervention and combination therapy.

GHs can selectively hydrolyze glycosidic bonds and eliminate dysfunctional glycans. Glycosylated prodrugs, which have been widely used to reduce the side-effects of anticancer drugs, can be activated through targeted deglycosylation mediated by certain GHs. One such enzyme, β-glucuronidase, has been utilized to cleave the glycans of prodrugs to activate them (53, 235). Various GHs, including glucosidase II (90, 236), FUCA2 (86), NEU1 (83), and NEU3 (85), are overexpressed in lung cancer and have the potential to be utilized in the development of lung cancer-specific prodrugs.

## 6 Conclusion and future perspective

CRDs encompass a range of inflammatory conditions affecting the respiratory tract. During inflammation reactions, various molecules involved in inflammation, such as p53 and selectins, undergo dysglycosylation. Targeting these glycoproteins represents a promising approach to anti-inflammatory and immunomodulation therapies. Moreover, the regulation of glycoenzymes and protein glycosylation by proinflammatory cytokines suggests a complex signaling pathway underlying the development and progression of CRDs.

Aberrant protein glycosylation plays a significant role in the pathogenesis of CRDs. Changes in the expressions of glycoenzymes in airway epithelial cells and mucus are responsible for variations in glycosylation patterns. Therefore, proteins with altered glycosylation patterns and various glycoenzymes present in the epithelial cells are potential targets for new monotherapies and combination therapies for CRDs. Abnormal protein glycosylation and glycoenzymes in body fluids, especially in sputum and serum, may serve as potential biomarkers for the diagnosis, prognosis, and treatment assessment of CRDs.

Glycoproteomics and glycomics are essential strategies for unveiling the protein glyco-codes, including glycosites, glycan structures, and glycosylation levels. This review focuses the glycoenzyme-protein glycosylation-CRD axis, highlighting two main aberrant terminal glycosylation modifications, fucosylation and sialylation, and their respective enzymes, FUTs and STs, in CRDs. The clinical potential of these glycoenzymes and glycosylated proteins has already been demonstrated in the diagnosis and treatment of CRDs.

The involvement of glycoenzyme-mediated glycosylation changes in CRD development and exacerbation suggests that targeting glycoenzymes and glycoproteins using chemical and biomacromolecular drugs may be a promising approach for CRD therapy. It is common to observe glycosylation changes such as increased fucosylation and sialylation. One potential avenue for treating different CRDs is inhibiting specific FUTs and STs. Furthermore, the study of GHs under different conditions can provide new insights into designing prodrugs that can minimize their side-effects. Despite some efforts to investigate glycoproteomic and glycomic changes in CRD research, there is still a considerable amount of work that needs to be done in the field of CRD development and diagnosis. A comprehensive understanding of the pathophysiological protein glycosylation in humans is urgently required. Therefore, a systematic mapping of such glycosylation patterns is essential to further advance our knowledge in this area.

## Author contributions

WC conceived and designed the review; XX and SK contributed to the literature collection and the art work. XX, SK, and WC wrote and revised the manuscript. All authors contributed to the article and approved the submitted version.
